# Locoregional Therapies and Remodeling of Tumor Microenvironment in Pancreatic Cancer

**DOI:** 10.3390/ijms241612681

**Published:** 2023-08-11

**Authors:** Maria Caterina De Grandis, Velio Ascenti, Carolina Lanza, Giacomo Di Paolo, Barbara Galassi, Anna Maria Ierardi, Gianpaolo Carrafiello, Antonio Facciorusso, Michele Ghidini

**Affiliations:** 1 Oncology Unit 1, Veneto Institute of Oncology IOV-IRCCS, 35128 Padua, Italy; mariacaterina.degrandis@iov.veneto.it (M.C.D.G.); giacomo.dipaolo@iov.veneto.it (G.D.P.); 2 Postgraduate School of Diagnostic and Interventional Radiology, University of Milan, 20122 Milan, Italy; velio.ascenti@gmail.com (V.A.); carolinalanza92@gmail.com (C.L.); 3 Oncology Unit, Fondazione IRCCS Ca’ Granda Ospedale Maggiore Policlinico, 20122 Milan, Italy; barbara.galassi@policlinico.mi.it (B.G.); michele.ghidini@policlinico.mi.it (M.G.); 4 Radiology Unit, Fondazione IRCCS Ca’ Granda Ospedale Maggiore Policlinico, 20122 Milan, Italy; annamaria.ierardi@policlinico.mi.it (A.M.I.); gianpaolo.carrafiello@policlinico.mi.it (G.C.); 5 Department of Oncology and Haemato-Oncology, University of Milan, 20122 Milan, Italy; 6 Section of Gastroenterology, Department of Medical and Surgical Sciences, University of Foggia, 71122 Foggia, Italy

**Keywords:** pancreatic cancer, locoregional treatments, tumor microenvironment, ablation therapies, radiotherapy

## Abstract

Despite the advances made in treatment, the prognosis of pancreatic ductal adenocarcinoma (PDAC) remains dismal, even in the locoregional and locally advanced stages, with high relapse rates after surgery. PDAC exhibits a chemoresistant and immunosuppressive phenotype, and the tumor microenvironment (TME) surrounding cancer cells actively participates in creating a stromal barrier to chemotherapy and an immunosuppressive environment. Recently, there has been an increasing use of interventional radiology techniques for the treatment of PDAC, although they do not represent a standard of care and are not included in clinical guidelines. Local approaches such as radiation therapy, hyperthermia, microwave or radiofrequency ablation, irreversible electroporation and high-intensity focused ultrasound exert their action on the tumor tissue, altering the composition and structure of TME and potentially enhancing the action of chemotherapy. Moreover, their action can increase antigen release and presentation with T-cell activation and reduction tumor-induced immune suppression. This review summarizes the current evidence on locoregional therapies in PDAC and their effect on remodeling TME to make it more susceptible to the action of antitumor agents.

## 1. Introduction

According to the Global Cancer Observatory, in 2020, pancreatic cancer ranked as the 12th most common cancer for incidence and the 7th in terms of annual number of deaths worldwide [1]. Incidence and mortality have shown an increasing trend in recent years [2], and pancreatic cancer will become the second cause of cancer-related death by 2030 in the United States [3]. At diagnosis, no more than 15–20% of patients are eligible for upfront surgery, while approximately 30–40% present with borderline resectable or locally advanced disease, and the rest of the patients are diagnosed with metastases [4].

The treatment of resectable pancreatic ductal adenocarcinoma (PDAC) involves surgery followed by adjuvant chemotherapy. Preferred regimens include Gemcitabine-Capecitabine and mFOLFIRINOX for patients with good performance status [5,6]. For patients with borderline resectable PDAC, various treatment regimens have been tested, but currently, there is still no consensus on the optimal therapeutic approach [7,8,9,10]. For advanced disease, first-line chemotherapy options for patients in good overall condition include gemcitabine plus nab-paclitaxel or FOLFIRINOX, as an alternative to gemcitabine alone [5,11,12].

Despite the advances made in treatment, the prognosis of PDAC remains poor, with a median overall survival (OS) of approximately 12 months for metastatic disease at diagnosis [5,12]. PDAC is an aggressive tumor, and the fact that it is often diagnosed at advanced stages limits the prospects for treatment. Additionally, tumor cells frequently exhibit mechanisms of resistance to available treatments, reducing the effectiveness of drugs [13]. Therefore, the development of novel therapeutic strategies is a major challenge. The efficacy and feasibility of locoregional treatments are currently under investigation. Local approaches such as radiation therapy, hyperthermia, microwave or radiofrequency ablation, irreversible electroporation, and high-intensity focused ultrasound exert their action on the tumor tissue, limiting toxicity to healthy tissues and altering the composition and structure of the tumor microenvironment (TME), potentially enhancing the action of other anticancer agents.

This review aims to summarize the current evidence on locoregional therapies in PDAC and their effect on remodeling the tumor environment to make it more susceptible to the action of antitumor agents.

## 2. Tumor Microenvironment in PDAC

**Figure 1:**
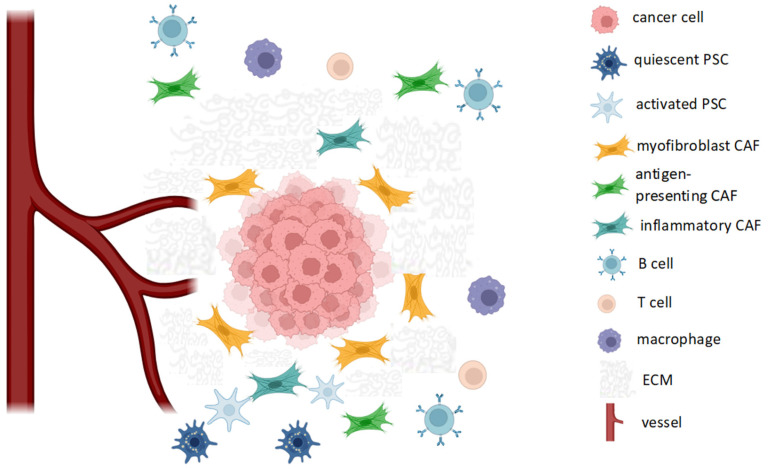
Schematic cellular composition of tumor microenvironment (TME) in PDAC. Tumor fibrosis is sustained by the action of pancreatic stellate cells (PSCs), which are activated from quiescent PSCs, as well as myofibroblast cancer-associated fibroblasts (CAFs). CAFs also play immunomodulatory roles (inflammatory and antigen-presenting CAFs). The immune-cell infiltrate is heterogeneous and includes B, T cells and tumor-associated macrophages. ECM: extracellular matrix.

The biological behavior of PDAC is strongly dependent on its interaction with the adjacent tissues. TME refers to all the normal cells, molecules and blood vessels that surround cancer cells. TME may be considered as a dynamic network of cells and stroma constituents; thus, its composition and functions are extremely various and it shows both an intratumor and intertumor heterogeneity [14,15] (Figure 1).

The cross talk between cancer cells and TME is responsible for tumor growth, metastatic potential and therapeutic resistance [16,17,18,19]. PDAC TME is made of various cell types: pancreatic stellate cells (PSCs), cancer-associated fibroblasts (CAFs), myeloid cells, regulatory T cells and B cells, endothelial cells and neuronal cells, which contribute to the formation of a tumor microenvironment characterized, among other things, by fibrosis, hypoxia and immunosuppression. Therefore, resistance to antitumor agents is sustained not only by intracellular mechanisms of the tumor cell (such as the lack of intracellular transporters) but also by the presence of a physical barrier that limits drug delivery [20]. Both PSCs and myofibroblast CAF contribute to the abundant production of extracellular matrix (ECM) molecules such as collagen, fibronectin, proteoglycan, that lead to fibrosis and high-grade tissue stiffness [21,22] (Figure 1).

PSCs are star-like-shaped cells that normally exist in a quiescent state but can be activated by various stimuli such as reactive oxygen species (ROS), cytokines, hypoxia and growth factors. When activated, PSCs can release components of extracellular matrix (ECM), metalloproteinases and maintain their activation through a loop sustained by the secretion of autocrine cytokines [23,24].

The Sonic Hedgehog (Shh) signaling pathway is often hyperactivated in many solid tumors, including PDAC, and appears to promote the activation of PSCs and desmoplasia [25,26]. In a murine model of PDAC, Shh inhibition through cyclopamine has been associated with improved survival [27]. Some trials have, therefore, assessed the role of Shh inhibitors in combination with chemotherapy in patients with advanced PDAC, yielding unsatisfactory results [28,29].

Furthermore, TGF-β, a pleiotropic cytokine that performs various functions, both tumor-promoting and antitumoral, is also involved in the deposition of ECM components, including fibronectin and collagens. TGF-β promotes the activation and proliferation of PSCs, which, in turn, release additional TGF-β through a positive feedback mechanism [30,31]. The use of TGF-β inhibitors has been evaluated in preclinical models and in some clinical studies, including a phase II study that compared gemcitabine plus galunisertib (an inhibitor of type I TGF-β receptor) versus gemcitabine plus placebo. The addition of galunisertib has been shown to increase survival, with a favorable tolerability profile [32]. CAFs are derived from activated PSCs, quiescent resident fibroblast and mesenchymal stem cells. They display distinct functions according to which we can distinguish myofibroblast CAFs that produce stroma, inflammatory CAFs that release cytokines involved in immune response, and antigen-presenting CAFs that are able to process and present antigens through the MHC-II complex [33,34]. 

Dense desmoplasia is a hallmark of the PDAC microenvironment and, together with the increased fluid pressure partly due to proteoglycan and hyaluronan, alters the organ architecture and represents an important obstacle to the delivery of therapeutic agents [35,36,37,38]. Moreover, the stroma of PDAC shows paucity of vascularity which similarly limits the diffusion of drugs [39,40]. Hypoxia and desmoplastic reaction support each other. In hypoxic conditions, hypoxia-inducible factor 1 alfa subunit (HIF-1α), unstable in normal oxygenation conditions, translocates in the nucleus, promoting the transcription of a variety of genes (including proangiogenic factors such as VEGF), thus creating a pro-inflammatory microenvironment. In response to such environment, PSCs are activated into myofibroblast-like cells. PSC under hypoxia increase the secretion of type I collagen, periostin and fibronectin [41,42], and hypoxia helps the release of Sonic Hedgehog ligand whose pathway promotes the secretion of type I collagen and fibronectin as well [43]. Furthermore, prolonged hypoxia is demonstrated to promote autophagy by HIF-1α and AMPK pathways in PSCs, thus decreasing production of inhibiting factors such as lumican (an extracellular matrix protein both secreted and present in PSCs cytoplasm), known for slowing PDAC cells proliferation [44,45,46]. Considering the role of hyaluronic acid in causing an increase in interstitial pressure, leading to vascular collapse and reduced perfusion, some trials have evaluated the addition of recombinant human hyaluronidase (PEGPH20) to chemotherapy in patients with metastatic PDAC. However, these trials did not show a better outcome despite increased toxicity [47,48].

**Figure 2:**
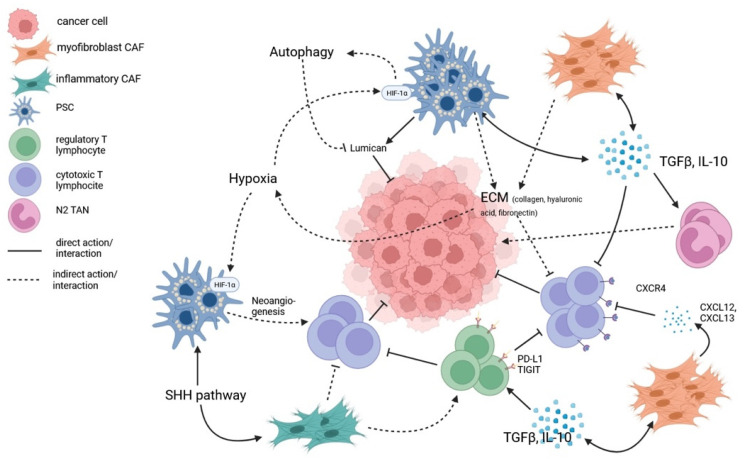
Schematization of key molecular interactions in PDAC microenvironment. Pancreatic stellate cells (PSCs) and cancer-associated fibroblasts (CAFs) secrete immunosuppressive cytokines (IL-10, TGFβ), furthering their proliferation through positive feedback, polarizing tumor-associated neutrophiles (TANs) towards their pro-tumorigenic N2 phenotype and inhibiting cytotoxic T lymphocytes (CTLs) function. Additionally, the deposition of extracellular matrix (ECM) and the overproduction of adhesion molecules ligands (CXCL12, CXCL13) impede normal lymphocytes motility, promoting immune evasion. Abundancy of ECM, moreover, facilitates hypoxia, which activates the HIF-1α pathway. This, on one hand, induces autophagy in cancer-inhibiting cell populations, but, on the other hand, promotes neoangiogenesis and CTLs infiltration.

PDAC exhibits an immunosuppressive phenotype, and the TME actively participates in creating an immunosuppressive environment by triggering mechanisms that promote immune evasion and restrict the activation of an effective antitumor immune response. CAFs may inhibit cytotoxic T lymphocyte (CTL) function with the release of immunosuppressive cytokines such as IL-10 and TGFB [49,50]. CAFs also restrict the movement of CTLs to the peri-tumoral stromal compartments through the activation of focal adhesion kinase (FAK) and the overproduction of C-X-C Motif Chemokine Ligand 12 (CXCL12), which binds the C-X-C Motif Chemokine Receptor 4 (CXCR4) [51]. Additionally, it has been demonstrated that, in the early stages of carcinogenesis, an infiltration of cells that facilitate mechanisms of immune evasion can be observed, such as tumor-associated macrophages (TAMs), myeloid-derived suppressor (MDSCs), and regulatory T cells (Tregs) [52,53]. Inhibition of the Hedgehog pathway leads to a reduction in the proportion of myCAF and an increase in inflammatory CAFs, resulting in a modification of the inflammatory infiltrate (reduction of CD8 T cells and an increase in regulatory T cells) [54] (Figure 2).

## 3. Role of Locoregional Therapies in PC and Effects on TME

Given the clinical need to improve treatment efficacy in PDAC, the development of new therapeutic strategies should take into consideration the important role of TME in supporting tumor growth and promoting treatment resistance. In this regard, locoregional treatments could reshape the TME, sensitizing tumor cells to systemic treatments by enhancing the delivery of cytotoxic agents or altering the composition of the immune infiltrate.

Microwave ablation (MWA), radiofrequency ablation (RFA), radiation therapy (RT), irreversible electroporation (IRE), high-intensity focused ultrasound (HIFU), and intra-arterial infusion chemotherapy are becoming increasingly popular due to their possibility to specifically target the tumor while limiting adverse events [55].

### 3.1. Ablation Therapies

Nonmetastatic locally advanced pancreatic cancer (LAPC) includes 30% of all newly diagnosed PDAC [56]. Standard-of-care treatment for LAPC is chemotherapy with or without radiation therapy. Neoadjuvant chemotherapy can downstage 20% of LAPC, leading to potential resectability with a significantly improved survival (35.3 months (mo)) compared to patients who do not become surgical candidates after treatment (16.2 mo) [57]. Patients with LAPC who do not become surgical candidates may benefit from local ablation therapies.

Locoregional ablative therapies are minimally invasive techniques that use a generator and a needle-like electrode to transmit energy directly to the target location, with the aim of producing tissue necrosis. These interventions are often performed percutaneously with the insertion of an electrode under imaging guidance; however, they can also be performed during laparoscopic/open surgery or endoscopy. The variety of minimally invasive ablative treatments may be classified according to their use of thermal energy, such as radiofrequency (RF), microwave (MO), and cryoablation (CA), or non-thermal energy, such as irreversible electroporation (IRE) [58]. High-Intensity Focused Ultrasound (HIFU) technology is another minimally invasive ablative method that, unlike the others described above, does not entail needle-like electrodes at the target tissue [59].

#### 3.1.1. Radiofrequency Ablation

In RFA, one or more electrodes create alternating currents at high frequencies, which cause very high local temperatures, resulting in thermal coagulation, protein denaturation and, consequently, thermo-coagulative necrosis of the target tissue. An ablated spherical area, generally 2 to 5 cm in diameter, is generated in about 10 to 30 min [60]. With RFA, the tissue heating zone is limited to a few millimeters surrounding the active electrode due to the high flow of electrical current, while the rest of the ablation zone is heated by thermal conduction [61]. Therefore, when the size of the target area increases, treatment efficacy is reduced, with a maximum result for volumes less than 3.5 cm [62]. This method of heat generation is dependent on conductivity with a close correlation to the water content of the tissue [63,64].

So far, there have been no randomized controlled trials (RCT) regarding RFA effectiveness. However, there is one ongoing RCT (PELICAN RCT) that aims to evaluate if the combination of chemotherapy and RFA prolongs OS compared to chemotherapy alone in patients with LAPC with absence of progression following two months of systemic treatment [65]. In a nonrandomized study, Giardino et al. enrolled 107 patients and divided them equally into two groups: group 1 underwent RFA as a first treatment, while group 2 received neoadjuvant therapy followed by laparotomic RFA. Twenty-nine patients also received intra-arterial chemotherapy (epirubicin and cisplatin into the celiac trunk). The median OS was 14.7 mo in group 1 and 25.6 mo in group 2. Patients treated with a combination of RFA, neoadjuvant therapy and intra-arterial chemotherapy had a prolonged median OS of 34 mo. Adverse events were reported in 25% of the cases [66,67]. In a recent systematic review on RFAs performed with endoscopic ultrasound (EUS) guidance, Spadaccini et al. evaluated a total of 120 patients from 14 studies with primary endpoints of adverse events and mortality. The pooled analysis showed a success rate of 99%, with an adverse events rate of 8% without mortality related to the procedure [68]. D’onofrio et al. reported their experience on percutaneous RFA of LAPC. They evaluated 30 patients with nonresectable PDAC nonresponsive to first-line chemotherapy treated with RFA percutaneously. At 30-days follow-up, no adverse events were reported. The mean survival was 310 days (65–718) [69].

Studies on PDAC treated with RFA are mostly focused on treatment via laparotomy or endoscopy, with only a small proportion of treatments performed percutaneously. When percutaneous RFA is feasible, it may avoid laparotomy, thus reducing the risk of infection, bleeding and other surgical complications [70]. In addition, while surgery involves impaired immune response, it is known that, in patients treated percutaneously, there is an enhanced immune system activation [71,72]. 

RFA can cause stroma denaturation and tissue permeability modifications in order to implement drug delivery. Ware et al. showed that RF affects molecular transport in a 3D model of PDAC with higher diffusion of DAPI fluorescence in spheroids in comparison to no RF. This could influence the response to medications that are passively diffusing in the TME, since the drug molecules would be able to extravasate in large quantities from the vasculature and disseminate deeper and more uniformly throughout the tissue [73].

RFA has been shown to induce a modification in the composition of the inflammatory infiltrate both in the treated tumor areas and in nontarget lesions distant from the locally treated zone, known as the “abscopal effect”. The “abscopal effect” refers to the response in a nontarget lesion distant from the treated site, which occurs due to immune activation and increased immune-responsiveness [74].

Faraoni et al. [75] used murine models with PDAC tumors, distinguishing between lesions treated with RFA and those without RFA treatment. Their results showed that RFA reduced PDAC tumor progression in vivo and favored a strong remodeling of the tumor microenvironment (TME), with an associated abscopal effect observed in 87.5% of non-RFA-treated tumors. A key role in the immune response in non-treated sites seems to be played by tumor-associated neutrophils (TANs), whose intralesional infiltration increases, and they are polarized into an antitumor phenotype. RFA also increases the expression of chemokines such as CXCL12, CXCL12 and CXCL13, recruiting B and T cells. Furthermore, they found an increase in PD-L1 levels compared to the control, with higher levels in RFA-treated tumors compared to non-RFA-treated ones, where the trend was increasing, although not significant. Similarly, Fei et al. evaluated immunological changes in RFA-treated and non-RFA-treated tumors using murine models. In tumors subjected to RFA, an increase in activated CD4 and CD8 cells, macrophages expressing NOS2 (associated with antitumor properties), and dendritic cells promoting proliferation and differentiation of T cells was observed. On the non-RFA side, the infiltration of CD8+ T cells, measured at 3, 5 and 8 days after the procedure, initially showed an increase compared to the control, but then reduced from the 3rd day onwards, suggesting that an immune response occurs, but it is likely transient and not effective [76]. Lawrence et al. conducted serial biopsies of LAPC in human patients before and after treatment with RFA on three occasions. They observed an upregulation of the CD1E gene, which is involved in antigen presentation, in both patients compared to the baseline. In one patient, an increase in genes related to T cells and their cytolytic function was detected. In both patients, there were alterations in the expression of genes related to the activity of CAFs [77]. It is not clear whether these changes can be effectively exploited to enhance the antitumor action of treatments, but they serve as evidence that RFA induces diverse modifications not only at the treatment site but also involving various aspects of tumor immune regulation and its interaction with the microenvironment.

#### 3.1.2. Microwave Ablation

Similar to RFA, microwave ablation (MWA) causes target tissue necrosis with a thermo-coagulative process. The majority of the heat produced by MWA is caused by the excitation of polar water molecules, while ionic polarization has a significantly lower impact. MWA produces a greater zone of active heating in a shorter time in comparison to RFA, allowing a more uniform necrosis in the target lesion. The two primary frequency bands utilized are 915 and 2450 MHz, with the latter being the more frequently employed [78]. The theoretical advantages of MWA over RFA are various: first of all, the ability to treat a larger lesion because the area of necrosis created is larger; moreover, the procedure has a shorter procedural time, less influence of vaporization and carbonization mechanisms and reduced heat dissipation in the presence of surrounding blood vessels (reduced heat sink effect) [67]. Although microwave ablation is a well-established intervention in liver, kidney, lung and bone malignancies [79,80,81,82,83,84], studies regarding this technology in PDAC are very limited. Carrafiello et al. evaluated the effectiveness of MWA (45 W power, 915 MHz frequency) in 10 unresectable PDAC (5 with percutaneous and 5 with laparotomic approach). The follow-up was on average 9.2 months (3 to 16). One late major complication was observed in one patient (gastroduodenal artery pseudoaneurysm, successfully treated with endovascular embolization); two patients had pancreatitis resolved during the hospital stay. The one-year survival rate was 80% with improvements in quality of life (QoL) for all patients in the first 6 weeks after treatment [85]. Vogl et al. treated with percutaneous MWA (5–100 W power, 2450 MHz frequency) 22 unresectable PDAC. Tumors were in the pancreatic head in 17 (77.3%) patients and in the pancreatic tail in 5 (22.7%). MWA’s technical success rate was 100%. There were no significant adverse events reported. Only patients who did not receive further neoadjuvant treatments 10/22 were evaluated for local tumor progression (LTP). Out of the patients evaluable, LTP was detected in one case (10%) at 3-months follow-up [86]. Ierardi et al. examined the viability and safety of percutaneous MWA (100 W power, 2450 MHz frequency) in 5 LAPC situated in the pancreatic head. At follow-up CT performed at 1, 3 and 12 months, no major adverse events were reported. An improvement in QoL was observed in all patients despite a tendency to come back to preoperative conditions in the months following the procedure [87]. In the context of MWA, percutaneous approach was the most frequently employed for the treatment of PDAC, which likely contributed to the decreased complication rates. However, there were some differences in the rates of MWA complications across the evaluated studies. This result, in addition to the difference in patient selection, can also be partly explained by the different technology used in the aforementioned studies.

#### 3.1.3. High-Intensity Focused Ultrasound

High-Intensity Focused Ultrasound (HIFU) technology is a noninvasive technique that uses high-energy ultrasound waves to ablate a limited target volume with US or MRI guide. Focused ultrasound devices are made of a generator that produces ultrasound energy and a transducer that focuses the waves into a beam aimed at a well-defined target region. HIFU has a dual effect on tissues, inducing both thermal and mechanical damage. The thermal effect generated by the absorption of sound waves is different whether the dose of deposited energy is low or high. At low energies (<55 °C), the hyperthermia induced does not generate cell death but increases cell membrane permeability. At high temperatures (>55 °C), cell death is induced by coagulation necrosis [88]. The mechanical damage, on the other hand, includes radiation force, increased pressure and acoustic cavitation [89]. In contrast to other heat-based ablation technologies, HIFU does not require the usage of needle-like electrodes and does not require routine employment of anesthesia even if the use of antispasmodic and anxiolytic drugs can be used to minimize involuntary movements of the patient [90,91]. Due to its heating effects, the most commonly explored use of HIFU is thermal ablation.

Numerous large-volume studies have shown that HIFU has a considerable positive impact on patients’ QoL, with improved tumor responsiveness and low rate of adverse events. A recent meta-analysis conducted by Fergadi et al. evaluated 939 patients with PDAC. They assessed that HIFU combined with neoadjuvant chemotherapy is a safe strategy that increases OS and causes less discomfort when compared to chemotherapy alone [92]. Another study by Ning et al. evaluated 523 unresectable PDAC treated with gemcitabine + HIFU or gemcitabine only. The median OS of patients receiving HIFU combined with gemcitbine vs. gemcitabine alone was 7.4 vs. 6.0 mo (*p* = 0.002), without any severe complication related to HIFU reported [93].

Besides thermal ablation, another application of HIFU that is recently gaining attention is its use as a means to provide targeted drug delivery. This is possible since ultrasound administered at high intensity promotes the creation of transient openings in the cellular membrane, increasing cellular permeability. This process is known as sonoporation [94]. There are two main motivations for using nanoparticles in combination with ultrasound. Firstly, the mechanical effects of HIFU are amplified by nanoparticles, reducing the cavitation threshold during the generation of microbubbles, leading to more effective therapeutic applications [95]. Secondly, carrier particles themselves can be loaded with drug molecules in order to be ablated (i.e., “activated”) at the proper delivery location by administering selective HIFU to that area [96]. In vitro experiments using PDAC spheroids composed of DT66066 cancer cells and normal fibroblasts showed that the same dose of gemcitabine is less cytotoxic in the presence of fibroblasts, supporting the hypothesis that the microenvironment can negatively influence the action of chemotherapeutic drugs. Moreover, they reported that cavitation generated by HIFU increased gemcitabine delivery and its therapeutic efficiency attributable to an increased cell membrane permeability, the damage of the cell membrane, an enhanced drug intake through sonoporation or ultrasound thermal effects and the synthesis of reactive hydroxyl species (ROS) [97]. In a mouse model of PDAC, Li et al. employed pulsed HIFU (pHIFU) to increase doxorubicin penetration via ultrasound-induced cavitation. They discovered that, in comparison to controls, the concentration of doxorubicin increased up to 4.5-fold. Additionally, normalized doxorubicin concentration was linked to the cavitation metrics (P 0.01), demonstrating that persistent high cavitation increases the penetration of treatment [98]. In a phase I clinical trial by Dimcevski et al., 10 patients with LAPC were treated with pHIFU at low intensity in conjunction with exogenously administered microbubbles to facilitate cavitation. This treatment, in combination with gemcitabine, doubled the median OS of gemcitabine single agent (17.6 months versus 8.9 months) [99].

Regarding temperature-dependent drug delivery, in vitro and in vivo animal models showed that doxorubicin was released more quickly and concentratedly after HIFU treatment in conjunction with injection of temperature-sensitive liposomes. Another work by Liang et al. showed that temperature-sensitive cerasomes released drug molecules in their target area when the temperature has increased by 5 °C [89,100]. The use of HIFU for facilitating drug delivery has certain limitations that should be considered, such as its short duration of action and variable drug uptake associated with treatment. Moreover, the efficacy of successful delivery of nanoparticles using HIFU can vary significantly within heterogeneous tumors and from patient to patient, leading to dramatic differences in the penetration and uptake of drugs [101]. In addition to the aforementioned properties of HIFU, an immunomodulating role for this therapy has recently been proposed. Wang et al. evaluated blood samples of 15 patients before and after HIFU therapy revealing larger percentages of circulating CD3+ and CD4 T cells (in 66% of patients), an increased CD4+/CD8+ T-cell ratio, and increased NK cell activity [102]. These results were supported by a recent meta-analysis of 3022 clinical cases of PDAC that had been thermally ablated using HIFU. Furthermore, hyperthermia induces the upregulation of heat shock proteins (HSP), which, in turn, stimulate the host’s immune system, and pancreatic necrosis in areas subjected to HIFU ablation results in the accumulation of IL-1 and IL-2, which are implicated in immune regulation [103].

#### 3.1.4. Cryoablation

Cryoablation (CA) is a thermoablative technology that is based on multiple cycles of freezing and thawing that results in the development of intra- and extracellular ice crystals, osmotic pressure fluctuations, disruption of cells membrane, and eventually cellular death [56]. One of the advantages of cryoablation over different thermal ablation techniques is the possibility to monitor the ablation zone during the procedure. In fact, during freezing, water in the tissue undergoes transition from liquid to solid, forming an “ice-ball” that is visible on ultrasound, computed tomography and magnetic resonance imaging [67]. In addition, because the cooling of tissue and nerves provides an anesthetic effect, CA tends to be less painful than heat-based thermal ablation technologies and, therefore, could theoretically be performed safely with moderate sedation only [104]. Experiments in vitro have shown that the temperature in which irreversible cell death is present in PANC-1 cell line after a single exposure is −25 °C; if repeated freezing cycles are performed, cellular death is present even at higher temperatures [105,106]. Interestingly, a complete cell death was found even after a single freeze at −15 °C if performed in combination with chemotherapy (100 nM of gemcitabine or 8.8 μM of oxaliplatin) before the ablation [106]. As for MWA, the available data on PDAC are very poor in literature. Xu et al. explored the possibility of treatment with CA (36 percutaneous and 13 intraoperative) in conjunction with 125I seed implantation in 49 patients with LAPC (12 of whom had liver metastases). Simultaneous CA was carried out for liver metastases positioning additional cryoprobes with an intercostal approach. During a median follow-up of 18 mo (range of 5–40 mo), the median OS was 16.2 mo. Complete response was recorded in 20.4% of patients, partial response in 38.8% of patients, stable disease in 30.6%, and progressive disease in 10.2% (5/49) of patients [107]. Niu et al. evaluated 67 patients with stage IV (metastatic) PDAC divided into four treatment groups: 22 of them had chemotherapy, 36 underwent CA alone, 17 had immunotherapy alone, and 31 received both CA and immunotherapy. Compared to the cryotherapy (7 mo), immunotherapy (5 mo), and chemotherapy (3.5 mo) groups, the CA-immunotherapy group’s median OS was considerably longer (13 mo). They performed tests for the immunologic index before treatment, both in patients treated with cryoimmunotherapy and in the immunotherapy group. There were no differences in any immunologic index between the groups, and they found that patients with normal immune function had a higher survival rate compared to patients with reduced immune activity. Unfortunately, the evaluation of the immunologic index was not repeated after the treatment, so it is not possible to assess any alterations of the tumor microenvironment induced by cryoimmunotherapy [108].

#### 3.1.5. Irreversible Electroporation

Irreversible electroporation (IRE) is an innovative ablative method employed in the clinical treatment of LAPC that provides intratumorally high-voltage electric pulses, causing the death of tumor cells by destroying the cell membrane integrity, creating nanopores. Compared to conventional ablative modalities, cell death in IRE is based on electrical energy rather than thermal energy and has different advantages: it is not influenced by the “heat sink effect”, avoiding incomplete ablation due to the energy reduction caused by blood flow; it preserves the extracellular matrix of vasculature and shows better safety profiles next to vital structures, especially for vital nerves, vessels, and cavity structures [109]. The preservation of vessels, which aids in the passage of immune molecules or cells, may result in a higher immune response [110].

**Figure 3:**
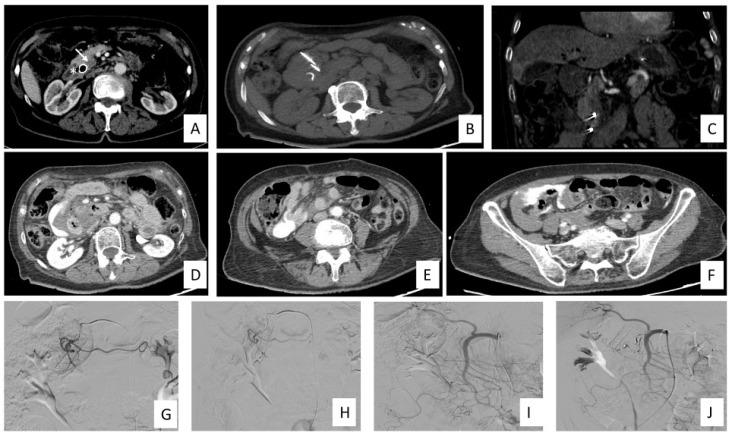
IRE for LAPC treatment timeline and complication management in a 76-year-old female patient. (**A**) CECT in arterial phase demonstrates the presence of the LAPC in the head of the pancreas (white arrows) and biliary stent (asterisk) prior to IRE treatment. (**B**) Axial view of noncontrast scan shows two needle electrodes in situ. (**C**) Coronal view in arterial phase shows the two electrodes in situ. (**D**–**F**) CECT scan immediately post procedure shows the presence of intraabdominal hematic fluid and extravasation of contrast media without any visible source of bleeding. (**G**–**J**) Angiography of celiac trunk, SMA, GDA, right renal artery and phlebography of the inferior vena cava and right renal vein did not demonstrate any source of bleeding. A preventive endovascular embolization of GDA and PDA were made using 3, 4 and 5 mm micro coils. Abbreviations: contrast-enhanced CT (CECT), irreversible electroporation (IRE), gastroduodenal artery (GDA), locally advanced pancreatic cancer (LAPC), pancreatic duodenal artery (PDA), superior mesenteric artery (SMA).

The procedure can be performed percutaneously under guidance (typically CT-guided), laparoscopically, or through an open approach following a midline laparotomy [111] (Figure 3). The percutaneous approach can be performed also with a transgastric approach [77,112].

Following the treatment, a contrast-enhanced CT scan should be done to confirm the correct ablation zone and to check for any early complications [77]. The mechanism of action is based on repeated cycles of brief, extremely high-voltage electrical pulses that change the transmembrane potential of tumor cells, causing the lipid bilayer of the cell membrane to develop nanoscale holes that increase membrane permeability. The membrane permeability becomes permanent under the right electrical conditions (90 pulses of 70 µs; electric field strength of 1500 V cm^−1^; delivered current of 20–50 A), and the cell dies due to loss of homeostasis [113]. The pulsatile application of electrical pulses at very high voltages poses particular difficulties for anesthesiologists, including the potential for inducing cardiac arrhythmias due to the tissue’s enhanced cell membrane permeability, which creates a pathway for ion transportation. Additionally, the activation of muscular or neurological tissue may result in strong muscle contractions and epileptic seizures. As a result, all IRE operations require general anesthesia and the use of neuromuscular blocking medications since total muscle paralysis is required to stop muscle contractions [113]. For these reasons, it is crucial to consider that several cardiac-related illnesses are absolute contraindications to this procedure [114]. The intensity of the electric pulses, which is inversely proportional to the distance between the electrodes and the tumor cells, determines the cytotoxicity of IRE. Additionally, the intratumoral heterogeneity may create low-pulse-strength areas where tumor cells can persist. In fact, insufficient ablation is frequently a cause responsible for local tumor recurrence after IRE [109].

It has been demonstrated that IRE not only destroys the tumor itself directly, but also has been shown to boost antitumor immunity and temporarily lessen the stroma-induced immunosuppression [109]. PDAC is known to have a microenvironment that is extremely immunosuppressive and has a low mutational burden, which results in a small number of neoantigens and permits the tumor to spread unhindered [115]. Compared to other ablation modalities, IRE might exhibit larger immune enhancing abilities in terms of protein release and T-cell activation compared to cryo- or heat ablation with enhanced antigen presentation, cause inflammation, and reduce tumor-induced immune suppression [115,116]. IRE induces a systemic immune response that results in the release of antigens and damage-associated molecular pattern molecules (DAMPs). These DAMPs are absorbed by dendritic cells (DCs) residing in tumor tissues; after that, the DCs go to draining lymph nodes where they become mature by the binding of toll-like receptor 9 on the plasmocytoid DCs. The DC maturation leads to the release of IFN-g and to the activation of cytotoxic and helper T cells that are specific for the tumor antigen, helping the development of a systemic immune response and therefore an active in vivo antitumor vaccination [115,117]. This systemic T-cell response could then lead to regression in distant metastases [74,114]. However, PDAC are well known for having a microenvironment that is extremely immunosuppressive, which makes it challenging for the proinflammatory T cells that have been activated to contribute to tumor elimination. A combination with immunotherapy in the form of checkpoint inhibitors or other active immune-enhancing therapies may have a synergistic impact to leverage the patients’ own activated immune system through the IRE technique [114]. O’Neill et al. tested the hypothesis that that IFN-g would cause expression of PD-L1 PDAC, and they performed an in vitro study where human pancreatic cell lines were cultured with interferon-g, and murine models of PDAC were treated with IRE, and PD-L1 expression was measured. They revealed that IRE induces expression of PD-L1 in vitro, and the combination therapy with concurrent nivolumab was well tolerated [118]. Zhao et al. [119], using murine models of PDAC treated with anti-PD1 or IRE or IRE + anti-PD1, demonstrated increased survival in those treated with the combination treatment. The authors highlighted an increase in CD8+ T cells in the group treated with IRE + anti-PD1 compared to the others, while no significant differences were observed in the frequency of CD4+ T cells, NK cells, B cells, DCs, or MDSCs among the groups. The same authors investigated the mechanisms of IRE-induced damage in an in vitro model and found that, at high voltages, there was an increase in the concentration of adenosine triphosphate (ATP) and high-mobility group protein B1 (HMGB1), known as damage-associated molecular patterns (DAMPs). Furthermore, IRE modulates the stroma by inducing necrosis in the tumor center, with an increase in microvascular density and reduced expression of HIF-1α, at the fourth day after the procedure, leading to increased blood vessel permeability, likely promoting the infiltration of cytotoxic lymphocytes. Additionally, several components of the tumor microenvironment were found to be downregulated, including FAPα, hyaluronic acid and LOX. FAPα+ CAFs produce CXCL12, which limits the intratumoral entry of lymphocytes. Hyaluronic acid, as previously discussed, increases interstitial fluid pressure, restricting the extravasation of immune cells, and LOX is implicated in the formation of a fibrotic network that restricts the infiltration of T cells. He et al. evaluated the role of IRE plus anti-PD-1 antibody versus IRE alone for patients with LAPC and demonstrated that IRE plus toripalimab had acceptable toxic effects and might improve survival in LAPC compared with IRE alone [110]. The tumor-associated neutrophils (TANs) can be divided into two phenotype groups: antitumor phenotype N and pro-tumor phenotype N2. Immunosuppressive molecules in the tumor microenvironment can polarize TANs into N2 phenotype, inducing the tumor activity. Recently, a tunable glutathione (GSH)-responsive mesoporous silica nanoformulation (dMSN-SB) was developed. It was shown to inhibit intratumoral TGF-𝛽 signaling, promoting TAN polarization toward the antitumor N1 phenotypes [109]. Peng et al. conducted a study demonstrating that dMSN-SB inhibits the TGFB signaling pathway and prevents the polarization of neutrophils into the pro-tumoral N2 phenotype in cell cultures. Subsequently, they investigated the use of dMSN-SB in combination with IRE and anti-PD1 in murine models and found that, after treatment, the infiltrate of CD8+ T cells was much more abundant compared to models treated with IRE + anti-PD1 agents or with IRE + dMSN-SB or the control. The group treated with the triple therapy showed lower expression of CXCL12, IL-1β, C5A, IL-1α, compared to the group treated with IRE + anti-PD1 [109].

### 3.2. Radiotherapy

Radiation therapy (RT) has been utilized in managing patients with PDAC both in the resectable and in the unresectable PDAC. Theoretically, preoperative radiation therapy has advantages over postoperative therapy, including better tissue oxygenation (which could increase RT effectiveness), sterilization of the operating field (which could reduce iatrogenic tumor seeding), and improved patient tolerance and compliance with treatment setting of resectable disease and unresectable disease [120]. The role of radiotherapy in unresectable LAPC is still debatable. RT has the potential to reduce the evolution of disease and potentially alleviate or avoid symptoms such as pain, biliary blockage, hemorrhage, and bowel obstruction. Nevertheless, the possibility of micrometastatic disease is significant, treatment is unlikely to be curative, and radiation can be toxic [121]. Recent advancements in RT techniques have resulted in an increased use of specific techniques including stereotactic body RT (SBRT), intensity-modulated RT (IMRT), and intraoperative RT (IORT). SBRT delivers 1 to 5 high-dose radiation fractions, while conventional RT delivers 25 to 28 fractions of 1.8/2 Gy each. The rationale behind SBRT is that tissues within the radiation field receive extremely high doses and are expected to suffer significant radiation-related damage. Some studies showed that SBRT in PDAC treatment is related to good local control rate but high rates of gastrointestinal toxicities [122,123,124]. In a recent series of 13 LAPC, a COMBO strategy was used, with a final SBRT boost given after initial chemotherapy first and subsequent concomitant chemoradiotherapy. The treatment strategy was well tolerated with good survival outcomes (median OS 21.5 mo, median PFS 17.5 mo) [125]. Intensity-modulated RT is delivered with conventional fractionation, but unlike conventional RT, the intensity of the radiation is nonuniform. Dose distribution is designed to minimize the radiation dose to normal tissues. Up to now, IMRT has not been proven superior to conventional three-dimensional RT, but IMRT techniques showed a reduction in dose to normal adjacent tissue during treatment of pancreas tumor [126,127]. Intraoperative RT (IORT) has been reached with the aim to increase radiation dose in the target tumor and, at the same time, to limit the radiation dose to adjacent normal structures such as bowel [121]. Although IORT is expected to improve local control, it has not been shown to increase survival. Consequently, despite the fact that IORT shows promise, the technique has not been widely used [128].

Different studies have investigated the role of RT in the immunomodulation in the human PDAC TME following therapy. Mills et al. evaluated the role of stereotactic body radiotherapy (SBRT) as a treatment modality for PDAC to prime cytotoxic T cells by inducing immunogenic tumor cell death in preclinical models [129]. They revealed that SBRT reduced PDAC cell density and induced immunogenic cell death (ICD) without damaging vasculature. However, the barrier to SBRT-induced antitumor immune responses in PDAC is an abundance of immunosuppressive myeloid populations. In conclusion, although SBRT may induce anticancer immune responses against human PDAC, the survival benefits are likely to be neutralized by long-term immune suppression mechanisms in the tumor microenvironment and future immunotherapy strategies are likely to be crucial to improve the treatment strategy.

### 3.3. Pancreatic Intra-Arterial Infusion Chemotherapy

Advanced PDAC has a very poor prognosis due to its chemoresistant nature. Chemoresistance of the tumor depends on the presence of a very dense and poorly vascularized fibrotic tumor stroma that involves tumor environment, the poor vascularization of PDAC and the high expression of the membrane-bound P-170 glycoprotein, part of an ATP-dependent drug efflux enzyme system [130].

Small case series have examined locoregional chemotherapy as an option for PDAC, demonstrating dose-dependent tumor sensitivity. In fact, when compared to systemic chemotherapy, pancreatic artery infusion (PAI) chemotherapy, using different chemotherapeutic agents such as gemcitabine and 5-fluorouracil, gives higher local concentrations of chemotherapeutic drugs while preserving healthy tissues and having a lower rate of side effects. Wang et al. evaluated the efficacy and safety of PAI with nab-paclitaxel in patients with advanced PDAC and demonstrated that PAI is safe, well tolerated, and effective for the relief of clinical symptoms [131]. Qiu et al. evaluated the efficacy and safety of PAI for the treatment of PDAC in patients unfit for chemotherapy or refractory, demonstrating that PAI is an effective and safe choice for this population, and, in addition, patients with a better performance status had better treatment outcomes [132]. Table 1 summarizes the available ablative treatments in PDAC.

## 4. Discussion and Future Perspectives

Apart from RT, locoregional treatments are not included in guidelines for the treatment of localized and advanced PDAC. Indeed, NCCN guidelines consider RT only in the case of PDAC amenable to receive preoperative treatment together with induction chemotherapy or alone (stereotactic body RT) before surgery. Moreover, in the case of LAPC, RT may be an option as a single treatment or together with chemotherapy [133]. However, in recent years, there has been an increasing use of interventional radiology techniques for the treatment of PDAC. In LAPC, interventional therapies must not be considered as an alternative to surgery. However, they may be used in patients unfit for or unwilling to undergo surgery or in the case of inadequate disease shrinkage after neoadjuvant treatment with subsequent impossibility to perform a radical resection. As far as advanced disease is concerned, locoregional treatments may be used as palliative strategies in patients with obstructive jaundice, liver metastases, in case of severe lower back pain and those who cannot tolerate systemic chemotherapy [134].

There is increasing evidence that locoregional treatments may remodel tumor TME and contribute to different mechanisms, as demonstrated through high-quality samples [135,136,137]. TME creates a stromal barrier to chemotherapy and actively participates in creating an immunosuppressive environment.

Locoregional treatments can modulate the composition of TME in multiple ways: on one hand, they promote structural alterations that facilitate the delivery of a higher amount of drug and, consequently, potentially enhance therapeutic efficacy. In this regard, several studies are evaluating the role of postlocoregional treatment chemotherapy for patients with nonmetastatic PDAC. In a phase II study, all patients undergoing IRE for the treatment of LAPC will receive either FOLFIRINOX or gemcitabine as peri-ablation treatment (NCT03484299). Another phase II study is determining the feasibility, tolerability, and treatment effect of EUS-RFA plus standard-of-care neoadjuvant chemotherapy with FOLFIRINOX or gemcitabine/nab-paclitaxel in LAPC (NCT04990609). A phase II/III trial has been completed, and patients were randomized to receive either IRE and synchronous chemotherapy with gemcitabine or IRE and subsequent gemcitabine starting on day 7 following ablative procedure in LAPC (NCT03673137). Differently, a phase I study is testing the tolerability of chemotherapy and EUS-RFA using the RF Electrode in patients receiving palliative second- or third-line therapy for unresectable nonmetastatic PDAC (NCT05723107). In addition, a phase II study has been completed and evaluated the safety and feasibility of the NanoKnife Low-Energy Direct Current (LEDC) System when used to treat unresectable PDAC (NCT01369420). On the other hand, locoregional therapies often modify the composition of immune response effectors, not only in the treated area, but sometimes also at a distance, as described in the abscopal effect promoted by ablative therapies. This leads to a polarization towards an antitumoral immune response with several observed modifications, including an increase in tumor-associated neutrophils (TANs) and the activation of B and T cells. Furthermore, some studies have documented an increase in PD-L1 expression following local treatments. These findings provide the rationale for the use of immune checkpoint inhibitors (ICI) in combination with locoregional therapies. In this direction, a phase II study is enrolling patients who will receive IRE for LAPC and will be treated with nivolumab postoperatively (NCT03080974). However, it should be noted that the duration and the actual antitumoral effectiveness of immune microenvironment modifications have not yet been established, and it remains to be seen whether they can be exploited therapeutically.

## 5. Conclusions

PDAC is a chemo- and immune-resistant disease with poor prognosis. Locoregional treatments, even if not considered standard of care, may have a role in interfering with TME with subsequent improvement of chemotherapy outcomes and increased immune sensitivity. Early phase and phase III studies are warranted in order to understand the impact of ablative therapies alone or in combination with chemotherapy/immunotherapy on disease response and survival outcomes.

## Figures and Tables

**Table 1:** Characteristics of ablative therapies in pancreatic cancer.

Technology	Mechanism of Action	Modalities of Intervention	Advantages	Disadvantages
*RFA*	Heat-based technology that produces coagulative necrosis through the creation of high-frequency alternating current	Laparotomy–Percutaneous– EUS	Economic Several long-term studies in literature	The use of ground pads is required. The ablation zone is limited to the immediate vicinity of the antenna.
*MWA*	Heat-based technology that generates coagulative necrosis through dielectric hysteresis of polar water molecules	Laparotomy– Percutaneous	It reaches remarkable ablation zones in a short time. A single antenna is usually sufficient.	There is little literature in the pancreatic field. Risk of damaging vascular structures
*HIFU*	High-frequency ultrasound-based technology that generates cellular damage either by temperature increase or through a mechanical damage	No needle placement is needed. US- or MRI-guided procedure	Ablation performed with very high degree of precision No placement of antennas required	Still not very widespread Limited treatment volume
*CA*	Cold-based technology that uses repeated freezing and thawing cycles to cause cell death	Laparotomy– Percutaneous	Real-time monitoring of the volume of the “ice ball” during treatment Less painful than heat-dependent treatment due to tissue and nerve cooling	Simultaneous use of multiple antennas often necessary Treatment is quite time-consuming.
*IRE*	Technology based on the use of high-voltage electrical pulses that allow the creation of nanopores in the cell membrane and subsequent cell death	Laparotomy– Percutaneous	Preserves surrounding structures like great vessels, bile ducts, and intestinal loops Systemic immune response against target tumor tissue potentially greater than other techniques	Expensive Need at least two needles parallel to each other to generate the circuit

Abbreviations: RFA: Radiofrequency Ablation; MWA: Microwave Ablation; HIFU: High-Intensity Focused Ultrasound; CA: Cryoablation; IRE: Irreversible Electroporation; EUS: Endoscopy Ultrasound.

## Data Availability

Data sharing not applicable.
